# A novel twin-grasper assisted mucosal inverted closure technique for closing large artificial gastric mucosal defects

**DOI:** 10.1007/s00464-023-10552-6

**Published:** 2023-11-20

**Authors:** Qinbo Cai, Huanjie Chen, Haobin Hou, Wenqing Dong, Lele Zhang, Minxuan Shen, Shaoxiong Yi, Rongman Xie, Xun Hou, Wentong Lan, Yulong He, Dongjie Yang

**Affiliations:** 1 Center for Gastrointestinal Surgery, The First Affiliated Hospital, Sun Yat-Sen University, Guangzhou, China; 2 Research Center for Diagnosis and Treatment of Gastric Cancer, Sun Yat Sen University, Guangzhou, China; 3 Laboratory of General Surgery, The First Affiliated Hospital, Sun Yat-Sen University, Guangzhou, China; 4 Department of Endoscopy, The First Affiliated Hospital, Sun Yat-Sen University, Guangzhou, China; 5 Digestive Diseases Center, The Seventh Affiliated Hospital, Sun Yat-Sen University, Shenzhen, China; 6 Guangdong Provincial Key Laboratory of Digestive Cancer Research, Shenzhen, China

**Keywords:** Large gastric mucosal defects, Closure technique, Wound healing, Endoscopic submucosal dissection

## Abstract

**Background:**

Large artificial gastric mucosal defects are always left unclosed for natural healing due to technique difficulties in closure. This study aims to evaluate the feasibility and safety of a new Twin-grasper Assisted Mucosal Inverted Closure (TAMIC) technique in closing large artificial gastric mucosal defects.

**Methods:**

Endoscopic submucosal dissection (ESD) was performed in fifteen pigs to create large gastric mucosal defects. The mucosal defects were then either left unclosed or closed with metallic clips using TAMIC technique. Successful closure rate and the wound outcomes were assessed.

**Results:**

Two mucosal defects with size of about 4.0 cm were left unclosed and healed two months after surgery. Thirteen large gastric mucosal defects were created by ESD with a medium size of 5.9 cm and were successfully closed with the TAMIC technique (100%), even in a mucosal defect with a width up to 8.5 cm. The mean closure time was 59.0 min. Wounds in eight stomachs remained completely closed 1 week after surgery (61.5%), while closure in the other five stomachs had partial wound dehiscence (38.5%). Four weeks later, all the closed defects healed well and 61.5% of the wounds still remained completely closed during healing. There was no delayed perforation or bleeding after surgery. In addition, there was less granulation in the submucosal layer of the closed wound sites than those under natural healing.

**Conclusions:**

The present study suggests that TAMIC is feasible and safe in closing large artificial gastric mucosal defects and could improve mucosal recovery compared to natural healing process.

**Supplementary Information:**

The online version contains supplementary material available at 10.1007/s00464-023-10552-6.

Endoscopic submucosal dissection (ESD) has been a widely accepted technique to remove superficial issues in gastrointestinal tract [1]. After ESD, artificial ulcers are created and whether these ulcers should be closed is controversial during past years [2, 3]. Without closure, artificial ulcers can naturally heal through the regeneration of epithelial cells and the formation of flat scars [4]. Recently, an increasing number of studies have shown that closing mucosal defects can reduce post-operative adverse events, such as delayed bleeding and perforation [5, 6]. Thus, endoscopists tend to close mucosal defects when there are risk factors of adverse events, such as small perforations or damage to the muscular propria during ESD [7].

Sometimes, extremely large artificial ulcers are created in the stomach. A gastric artificial ulcer larger than 4 cm often takes more than 3 months to heal [8], while closure of defects significantly speeds up the healing process [9]. It seems that closure of large mucosal defects can not only reduce adverse events but also prevent patient from experiencing long-term ulcers, which is crucial for their quality of life. However, closing defects smaller than 2 cm is easy, whereas closing large defects is challenging, and there is still no widely accepted technique for closing large mucosal defects [10]. Recently, we developed the Twin-grasper Assisted Mucosal Inverted Closure (TAMIC) technique and successfully closed large perforations after gastric endoscopic full-thickness resection (EFTR) [11, 12]. This study aims to evaluate whether this new technique can successfully close large artificial gastric mucosal defects.

## Materials and methods

### Animals and preoperative preparation

This study was approved by the animal experiment ethics committee of Silver Snake (Guang Zhou) Medical Technology Co., Ltd (Guangzhou, China, ss-2021-ZSYY). Thirteen live Tibetan pigs were obtained from Songshanhu Mingzhu Experimental Animal Technology (Dongguan, China), with a median weight of 38.5 kg (range 24.0–45.0 kg) (Tables 1, 2). The animals were provided a liquid diet for 3 days before the surgery to empty their stomachs. Anesthesia was induced with 3 mg/kg tiletamine and zolazepam (Zoletil, Virbac, France) via intramuscular injection, and 2 mg/kg propofol by intravenous injection. A 1.5% inhaled isoflurane was used to maintain anesthesia. Cefuroxime (40 mg/kg) was administered to the animal via intravenous drip 30 min before surgery. Physiologic parameters were monitored and maintained in a normal condition throughout the procedure.

**Table 1:** Individual characteristics and outcomes of the mucosal defects

ID	Gender	Age/months	Weight/kg	Lesion size/cm		ESD surgery time/min	Closure time/min	Clips number	Closure efficiency after surgery			Delayed bleeding	Delayed perforation	Submucosal abscess	Submucosal hematoma
				L	W				Week 1	Week 2	Week 4				
Natural healing															
1	Male	13	31.0	4.0	3.5	49	NA	NA	NA	NA	NA	No	No	No	No
2	Male	9	44.0	3.9	3.6	30	NA	NA	NA	NA	NA	No	No	No	No
TAMIC															
1	Male	13	40.0	2.8	2.6	20	10	11	Complete	Complete	Complete	No	No	No	No
2	Male	13	40.0	3.0	2.5	40	18	10	Complete	Complete	Complete	No	No	No	No
3	Female	10	39.5	4.4	4.1	24	59	15	Partial^a^	Partial	Partial	No	No	No	No
4	Female	10	38.5	4.0	3.7	29	42	15	Complete	Complete	Complete	No	No	No	No
5	Male	9	28.5	4.7	4.4	39	35	16	Complete	Complete	Complete	No	No	No	No
6	Male	9	27.5	5.5	5.3	46	63	23	Complete	Complete	Complete	No	No	No	No
7	Male	10	41.0	5.9	5.8	64	41	23	Partial^a^	Partial	Partial	No	No	No	No
8	Male	9	24.0	7.6	7.5	71	130	29	Complete	Complete	Complete	No	No	No	No
9	Female	10	30.5	6.5	6.2	65	106	27	Partial^a^	Partial	Partial	No	No	No	No
10	Female	11	28.0	6.3	6.1	55	76	22	Complete	Complete	Complete	No	No	No	No
11	Female	11	30.5	7.6	7.0	63	59	22	Complete	Complete	Complete	No	No	No	No
12	Male	10	45.0	8.5	8.4	67	55	31	Partial^a^	Partial	Partial	No	No	No	No
13	Female	10	44.5	7.5	7.3	106	64	29	Partial^a^	Partial	Partial	No	No	No	No

*L* length, *W* width

^a^Partial wound dehiscence (from middle to one side of the wound)

**Table 2:** Summary of lesion characteristics and technique outcomes (*n* = 13)

Characteristics/outcomes	Proportion (%, *n*/*N*)/median (range)	Mean ± SD
Body weight (kg)	38.5 (24.0–45.0)	35.2 ± 7.2
Sex		
Male	53.8% (7/13)	NA
Female	46.2% (6/13)	NA
Lesion size (cm)		
Length	5.9 (2.8–8.5)	5.7 ± 1.8
Width	5.8 (2.5–8.4)	5.5 ± 1.9
Complete closure rate	100.0% (13/13)	NA
Procedure time of ESD, min	55.0 (20.0–106.0)	53.0 ± 23.5
Procedure time of TAMIC, min	59.0 (10.0–130.0)	58.3 ± 32.7
Number of clips per perforation	22.0 (10.0–31.0)	21.0 ± 7.0

*NA* not applicable

### Surgery procedure

**Fig. 1:**
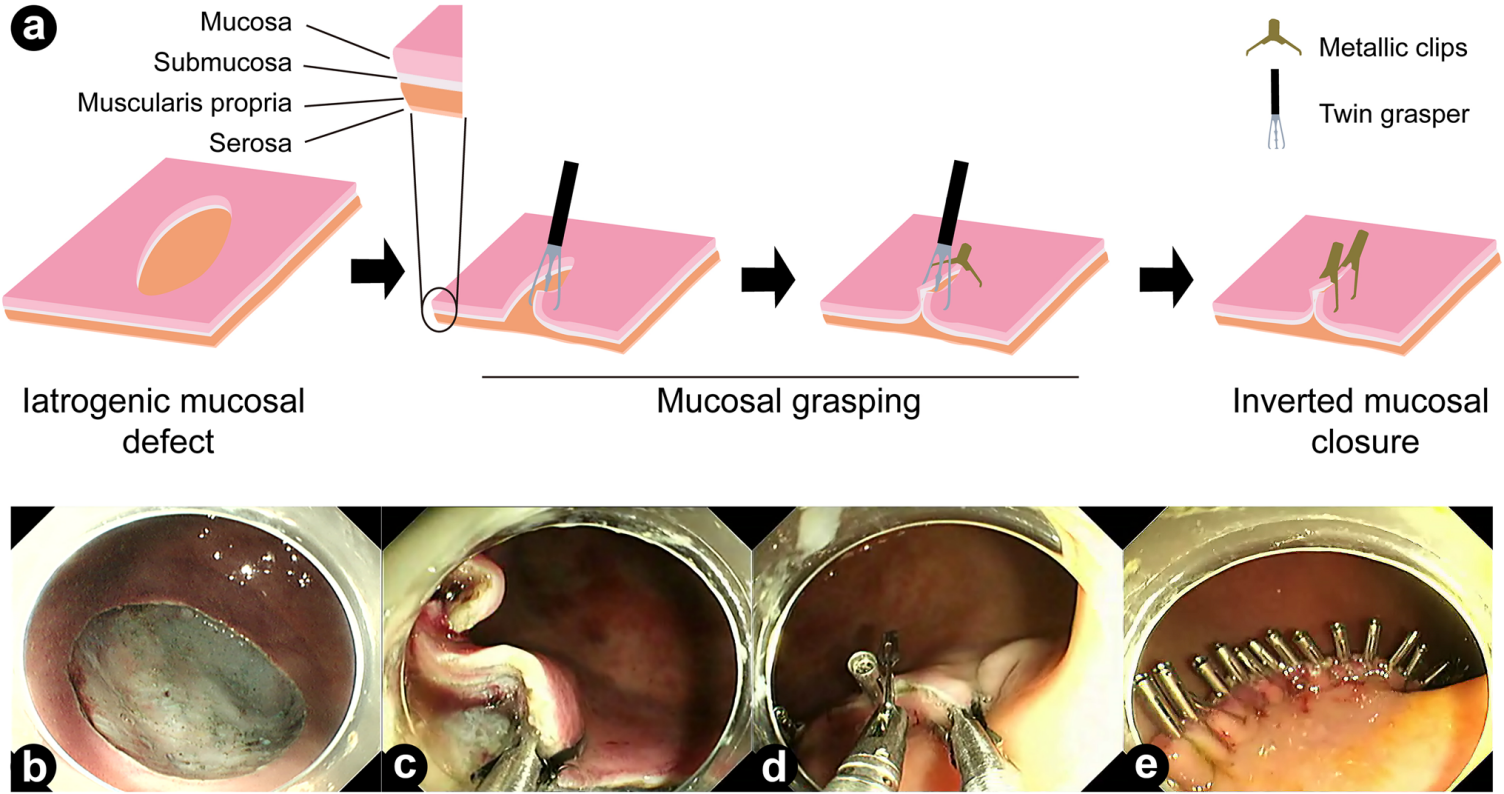
The procedure of the Twin-grasper Assisted Mucosal Inverted Closure (TAMIC) technique. **a** A diagram showing the procedure of the TAMIC technique. Briefly, mucosal layers in opposite sides were approximated tightly by the twin grasper under an endoscope with two working channels, and metallic clips were sent into another working channel to close the mucosal defect. **b–e** Representative images of TAMIC technique. A large artificial mucosal defect was created by endoscopic submucosal dissection (ESD) (**b**). One side of the mucosal layer was grasped by the twin grasper (**c**). Two sides of the mucosal defect were approximated by the twin grasper (**d**). The large mucosal defect was successfully closed by metallic clips with the mucosal layer in an inverted way (**e**)

All the mucosal defects were created in the body of posterior gastric wall and ESD was performed as previously reported [1]. For setting of the size of ESD, we used alcohol disinfected pig hair with given length. However, the exact size of the mucosal defects were measured according to sizes of the resected mucosal lesions (Fig. S1). Mucosal defects in two animals were left unclosed to observe the natural healing process and harvested for pathological analysis 14 weeks after surgery. For the closure of mucosal defects, an endoscope with two working channels (Olympus, Tokyo, Japan) and a twin-grasper (Ovesco, Tuebingen, Germany) were used, as previously reported [11, 12]. Briefly, mucosal layers in opposite sides were tightly approximated using the twin-grasper, and metallic clips (Micro-Tech, Nanjing, China) were then inserted into another working channel to close the mucosal layers (Fig. 1). All the procedures were performed by Dr. Dongjie Yang, a senior endoscopist with over 50 thousands diagnosed endoscopies and over 500 endoscopic surgeries.

### Post-ESD management and long term follow-up

Pigs were fasted for 1 week after surgery with parenteral nutrition (400 ml multiple electrolytes, 250 ml fat emulsion injection, 150 ml 5% glucose and 200 ml 0.9% sodium chloride injection). Then, a liquid diet was provided for another week, consisting of 400 g enteral nutritional powder (ENSURE, Abbott, the Netherlands) in 1800 ml of water per day. Cefuroxime (40 mg/kg/day) and Omeprazole (2 mg/kg/day) were administered for 1 week. Repeat gastroscopy was performed in week 1, week 2, and week 4 after surgery to examine the wound sites, and then the animals were sacrificed in week 4 after surgery. The primary outcomes were the successful TAMIC closure rate and the completely closed healing rate. Completely closed healing means there is no wound dehiscence during the healing process. Secondary endpoints were closure time, wound healing of the submucosal layer, and adverse events, including delayed bleeding and delayed perforation. Delayed bleeding was defined as melena or a reduction in hemoglobin by over 5 g/L from day 1 to week 4 after surgery. Delayed perforation was preliminarily judged based on reduced intake, fever, increased abdominal protuberance, and abdominal muscular tension from day 1 to week 4 after surgery.

### Statistics

Statistical analyses were performed using GraphPad Prism (version 9, GraphPad Software, San Diego, USA). Variables are presented as mean ± SD/median (range), or proportion, according to data type. Mann–Whitney *U* test was used to compare the difference of variables. Two-tailed tests and an α of 0.05 were used for all statistical analyses.

## Results

### The natural healing process of large mucosal defects

**Fig. 2:**
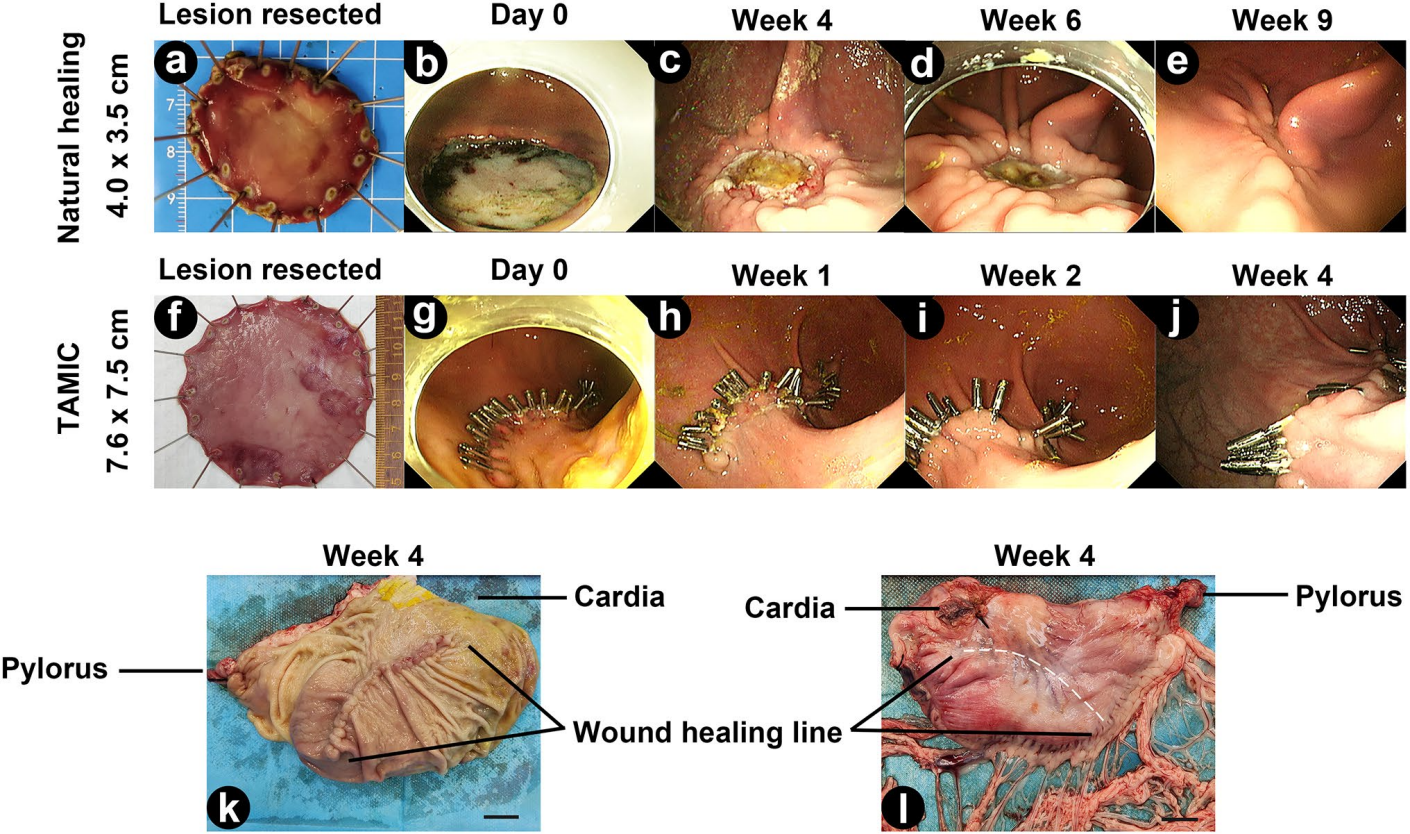
Representative images of the healing process of natural healing and TAMIC closure. **a** The mucosal lesion resected in natural healing group. **b–e** Overview of the wound site immediately after surgery (**b**) and 4 weeks (**c**), 6 weeks (**d**) and 9 weeks (**e**) after surgery. **f** The mucosal lesion resected in TAMIC group. **g–j** Overview of the wound site immediately after surgery (**g**) and 1 week (**h**), 2 weeks (**i**) and 4 weeks (**j**) after surgery. **k, l** Representative images of the mucosal and serosal wound sites after the stomach was harvested. White dot line indicating the wound healing line of the mucosal defects. Scale bar: 2 cm. *TAMIC* Twin-grasper Assisted Mucosal Inverted Closure (Color figure online)

In the pilot study, we performed ESD and resected two mucosal lesions with sizes of 3.9 and 4.0 cm to observe the natural healing process of large mucosal defects (Table 1). We found that natural healing of mucosal defects with size of 3–4 cm need more than 2 months and finally the wound site was replaced with a flat scar (Fig. 2a–e).

### TAMIC can successfully close large artificial gastric mucosal defects up to 8.5 cm

We performed ESD and resected thirteen mucosal lesions ranging in size from 2.8 cm to 8.5 cm as TAMIC group (Table 1). All the mucosal defects in TAMIC group were successfully closed using the TAMIC technique, with a medium operation time of 59.0 min (range 10.0 to 130.0 min). On average, 22.0 metallic clips were used for each defect (Table 2).

The first follow-up gastroscopy was performed 1 week after surgery, and the results showed that eight of the defects remained completely closed, with a majority of the metallic clips intact (61.5%) (Fig. 2), while five of the wounds had partial wound dehiscence (Fig. S2). The dehiscence occurred from the middle to one side of the wound (Fig. S2d and i). Two weeks after surgery, the closure conditions of all the defects were almost the same as those 1 week after surgery, and no further dehiscence was observed. Additionally, ulcer healing was apparent in the wound sites with dehiscence (Fig. S2e and j). Four weeks later, all thirteen pigs were sacrificed, and the stomachs were harvested. In the cases with sustained closed healing, there was a long mucosal scar in wound sites that remained completely closed, with little deformity in the inner cavity of the stomach (Fig. 2j, k). Interestingly, no deformity was found in the seromuscular layer (Fig. 2l). No adverse events were observed throughout the entire experiment, such as delayed bleeding or delayed perforation (Table 3). These results suggests that closure of large mucosal defects by TAMIC is safe and feasible.

**Table 3:** Summary of post-surgery outcomes (*n* = 13)

Outcomes	Proportion (%, *n*/*N*)
Sustained closed healing^a^	61.5% (8/13)
Delayed bleeding	0% (0/13)
Delayed perforation	0% (0/13)
Submucosal abscess	0% (0/13)
Submucosal hematoma	0% (0/13)

^a^Wound healing without any dehiscence or ulcer

To explore the possible risk factors for partial dehiscence of the mucosal layer, we summarized and compared all the potential risk factors between the partial dehiscence group and the completely closed healing group. However, no statistical significance was found among all the factors (Table S1).

### There is less submucosal granulation formation with no abscess or hematoma in the wound sites closed by TAMIC compared to those with natural healing

**Fig. 3:**
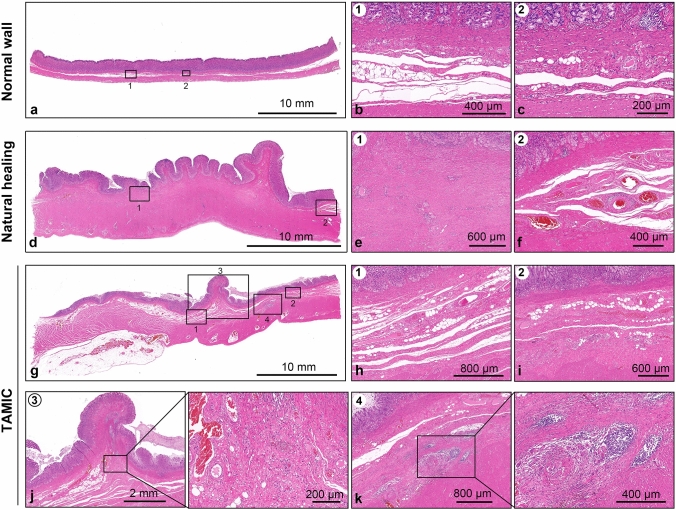
Granulation tissue in the submucosal layer upon the defect floor. **a** Representative normal gastric wall far away from the wound sites. **b** Normal submucosal layer with multiple loose space. **c** Normal submucosal layer with 1–2 loose space. **d** Representative image of gastric wall in the wound sites of the natural healing group. **e** Submucosal layer with sever granulation in the wound site. **f** Submusal layer with no granulation in the wound margin. **g** Representative image of gastric wall in the wound sites of the TAMIC group. Length of the seromuscular layer was similar as that of the mucosal layer. **h** Submucosal layer with multiple loose space similar to normal gastric tissues. **i** Submucosal layer with 1–2 loose space similar to normal gastric tissue. **j** Submucosal layer in the mucosal healing site. Little granulation formation right below the mucosal wound sites could be observed. **k** Submucosal layer with local immune cell infiltration. *TAMIC* Twin-grasper Assisted Mucosal Inverted Closure

To examine the wound healing of the submucosal layer in the defect sites, the entire stomach wall with the wound sites was fixed with 10% formalin and resected transversely at 0.5 cm intervals, and finally stained with hematoxylin–eosin (HE) staining. In this study, we observed apparent dead space during defect closure (Video S1). However, the results showed that the submucosal layers in the completely closed wound sites had similar loose connective tissues as those in the adjacent normal gastric wall (Fig. 3a–c), and there were no dead space, abscesses or hematomas in the submucosal layers 4 weeks after surgery (Fig. 3g–k). Only a small amount of granulation formation was observed just below the mucosal wound sites (Fig. 3j), which was much less than the natural healing process (Fig. 3d, e). However, there was local immune cell infiltration in some of the submucosal layers, which was absent in the normal mucosal layers (Fig. 3k). These results indicates that the closure of large mucosal defects by TAMIC is capable of restoring the normal anatomy of gastric wall.

## Discussion

In this study, we successfully closed thirteen large artificial mucosal defects using our recently developed TAMIC technique, and the 100% closure rate indicated the technical feasibility of TAMIC in closing large mucosal defects. Although five of the wounds (38.5%) failed to remain completely closed after 1 week of surgery, TAMIC somehow reduced the size and accelerated the healing process of large mucosal defect. Additionally, no adverse events were found during the experiments, which confirms the safety of TAMIC in closing large artificial ulcers.

Large gastric mucosal defects are always left as artificial ulcer and heal naturally, as the closure of large gastric mucosal defects is technologically challenging [13]. Traditional through-the-scope clips (TTSC) are only feasible for closing mucosal defects with a length less than 2 cm, mainly due to the limitation of the open width between the two tips [10]. In this study, we used a twin-gasper to approximate the two sides of large mucosal defects, and thus created condition for the closure of large mucosal defects simply with TTSCs. Besides using the most commonly available TTSCs, TAMIC also has two other advantages. Firstly, the relative movements of the twin-gasper and TTSC makes it easy for TTSC to clamp as much mucosal tissue as possible. Secondly, the twin-grasper ensures that the mucosal layers can be closed in an inverted way (Fig. 1), which prevents mucus secretion in the wound sites and might be a better condition for mucosal healing compared to the face-to-face condition of mucosal epithelial cells. Also, we want to highlight the function of the twin-grasper in dealing with the curly and sunk mucosal margins. By using the twin-grasper to readdress the curly and sunk margins in an inverted way, it was easily to clamp the mucosal margins and to avoid too much curly mucosal tissue exceeding the capacity of the clip (Video S1).Technically, TAMIC is similar to the over the scope clips (OTSC). However, OTSC is expensive and not commonly available, and it can only close mucosal defects with parameters smaller than 2 cm [14]. Endoloops or O-ring/loop 9 combined with TTSCs can theoretically close large mucosal defects. However, it is difficult for these techniques to approximate the mucosa tightly or in an inverted way [15–18]. It was recently reported that reopenable clip over line method (ROLM) could close extremely large gastric mucosal defect and avoid submucosal dead space. However, ROLM required special clips with holes for a line, and the two sides of the mucosal layer are not tightly approximated [19]. Through-the-scope twin clips (TTS-TCs) are also capable of approximating mucosal defects but may be difficult to clamp enough mucosal tissue without the assistance of other tools [20]. Compared to other techniques, it seems that TAMIC can provide tighter closure, allowing for the successful closure of large mucosal defects in this study.

In this study, we chose the posterior wall as the representative location for mucosal defects for the following reasons: First, this study aimed to evaluate the feasibility and safety of TAMIC. However, the location was supposed to be one of the most important factors affecting the outcomes. In order to gain a solid evidence with limited animals, we choose to do all the defects in a representative location first. Second, according to our experience, the posterior wall may be more fixed as part of the lesser omental bursa, which might cause higher tension during closure compared to the anterior wall. Thus, it seemed that TAMIC could also be successful in the anterior wall if we could get positive results in the posterior wall. However, we are going to do further studies to confirm this hypothesis in future. TAMIC might be challenging in the traditional technically difficult anatomic locations, such as gastric fundus and angularis, which we are also going to explore in future studies.

Dead space in the submucosal layers has been reported as one of the concerns when closing large mucosal defects [19]. In this study, we also observed apparent dead space (**Video S2**). However, there were no abscesses or hematomas in the submucosal layers in this study 4 weeks after surgery, which indicated that dead space might not be a risk factor for wound healing of large mucosal defects closed by TAMIC. There might be two reasons contributing to this satisfactory outcome. First, TAMIC provided tight enough closure and prevented gastric juice from entering the submucosa. Second, the submucosal layer of the stomach contains abundant blood vessels and immune cells, allowing for good absorption and anti-infection abilities. Both physical and histological examinations showed that the mucosal layer adhered well to the seromuscular layer in the wound sites. These results suggest one possible mechanism that the mucosal layer extends to a similar distance as the seromuscular layer. In the stomach mucosa, there is continuous regeneration through the differentiation and proliferation of stem or progenitor cells, which is responsible for self-renewal within days to months and provides enough epithelial cells for the extension of the mucosal layer [21]. This may be the cellular basis for the extension of the mucosal layer.

However, there were also five cases (38.5%) with partial dehiscence in this study. Although the risk factor analysis failed to find out statistically significant reasons, we found that the time for ESD surgery and TAMIC was longer in the partial dehiscence group than in the completely closed group (Table S1). According to our experience, longer operation time always results in severe mucosal edema and increased difficulty in closure. More repeated grasping by the twin-grasper also enhances mucosal damage and edema, creating a negative feedback loop. Therefore, a short time and high quality closure might be important for maintaining the completely closed healing.

In the natural process of regenerating an artificial ulcer, granulation tissue appears over the ulcer floor and eventually develops into a flat scar with regenerative gastric mucosa forming around the ulcer edge [13]. Stomachs with closed mucosal defects in this study showed no flat scar but little submucosal granulation, which is consistent with previous studies [9]. The formation of granulation tissue leads to ulcer scar contraction and convergence towards its center, ultimately contributing to mucosal deformity [4, 22, 23]. Thus, mucosal extension or relative movement will be impossible if there is aberrant granulation tissue over the defect floor. These results suggest that the closure of mucosal defects might be an alternative choice to prevent granulation tissue formation and, ultimately, prevent strictures from occurring in the pylori or gastroesophageal junction.

However, this study is a porcine research with several limitations. Firstly, porcine stomach is different from human stomach, with an apparently larger thickness of the wall. Studies in human stomachs should be conducted to confirm the results. Secondly, this study only created mucosal defects in the posterior gastric wall, and other sites should be tested in future studies. It may be difficult to perform TAMIC in the fundus and angle of the stomach, where reverse endoscopy is always needed to achieve better access. Finally, the small animal number limits our evaluation of the completely sustained closed healing rate and the possible risk factors. A statistically proved efficient number should be calculated based on this study in future research. The average procedure time of TAMIC seemed long in this study (average of 59.0 min), which was likely due to the difficulty in approximating the mucosal edges with the twin-grasper. The twin-grasper from Ovesco was unable to rotate, making it sometimes difficult to grasp the mucosal edge appropriately. Great tension also existed during approximating two sides of the large mucosal defects, and the mucosa sometimes slipped off the twin-grasper. Thus, practice and modification of the twin-grasper are both important to shorten the procedure time of TAMIC.

In conclusion, the present study suggests that the closure of large gastric mucosal defects by TAMIC is feasible and safe and could improve mucosal recovery compared to natural healing process. However, further studies in patients should be developed to confirm the clinic value of the TAMIC technique.

### Supplementary Information

Below is the link to the electronic supplementary material.

Video S1 (MP4 20703 KB)

Video S2 (MP4 60742 KB)

Supplementary file 1 (DOCX 4352 kb)
