# Supplementary material for: A novel twin-grasper assisted mucosal inverted closure technique for closing large artificial gastric mucosal defects
Source: Surg Endosc. 2023 Nov 20;38(1):460–8. doi: 10.1007/s00464-023-10552-6 (PMC10776692; doi:10.1007/s00464-023-10552-6)
Supplement: Supplementary file 3 — Supplementary file 1 (DOCX 4352 kb) [file 464_2023_10552_MOESM3_ESM.docx]

**Supplementary materials**

**Supplementary table**

| **Table S1** Risk factors for partial wound dehiscence. | | |  |
| --- | --- | --- | --- |
| **Factors** | **Partial dehiscence**  **Median (range)/Mean ± SD**  **(n = 5)** | **Complete closed healing**  **Median (range)/Mean ± SD**  **(n = 8)** | ***P* value** |
| **Leision size**, cm |  |  |  |
| Length | 6.5 (4.4 - 8.5)/6.6 ± 1.6 | 5.1 (2.8 - 7.6)/5.2 ± 1.9 | 0.27 |
| Width | 6.2 (4.1 - 8.4)/6.4 ± 1.6 | 4.9 (2.5 - 7.5)/4.9 ± 1.9 | 0.22 |
| **ESD operation time**, minutes | 65.0 (24.0 - 106.0)/65.2 ± 29.0 | 43.0 (20.0 - 71.0)/45.4 ± 17.1 | 0.17 |
| **Closure time**, minutes | 59.0 (41.0 - 106.0)/65.0 ± 24.5 | 50.5 (10.0 - 130.0)/54.1 ± 38.0 | 0.55 |
| **Number of clips/leision size** (per cm) | 3.9 (3.4 - 4.2)/3.8 ± 0.31 | 3.7 (2.9 - 4.2)/3.6 ± 0.4 | 0.35 |

**Supplementary figures and figure legends**


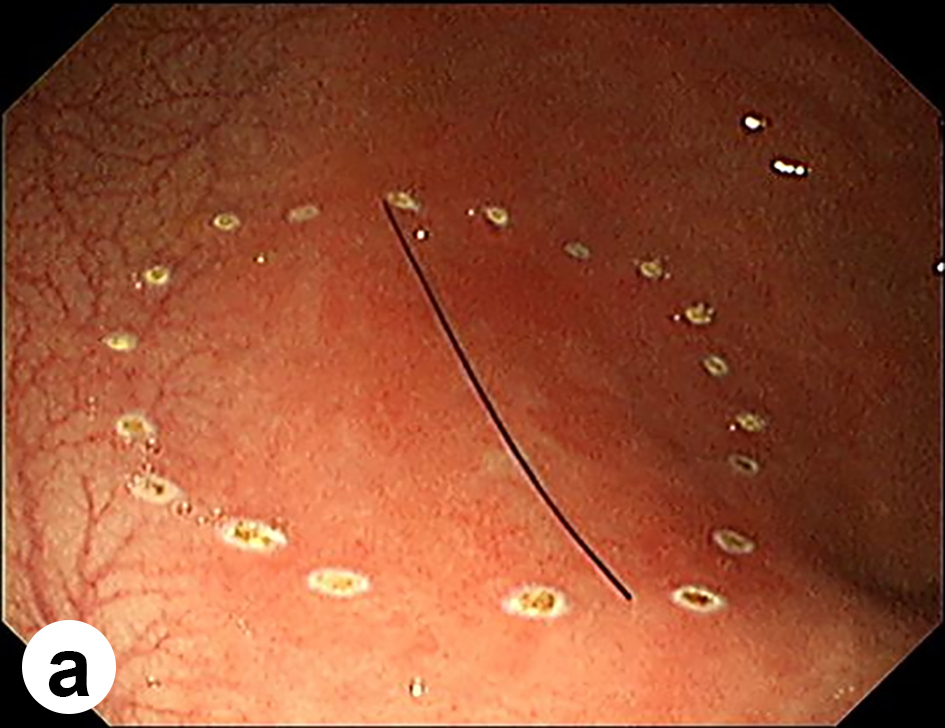

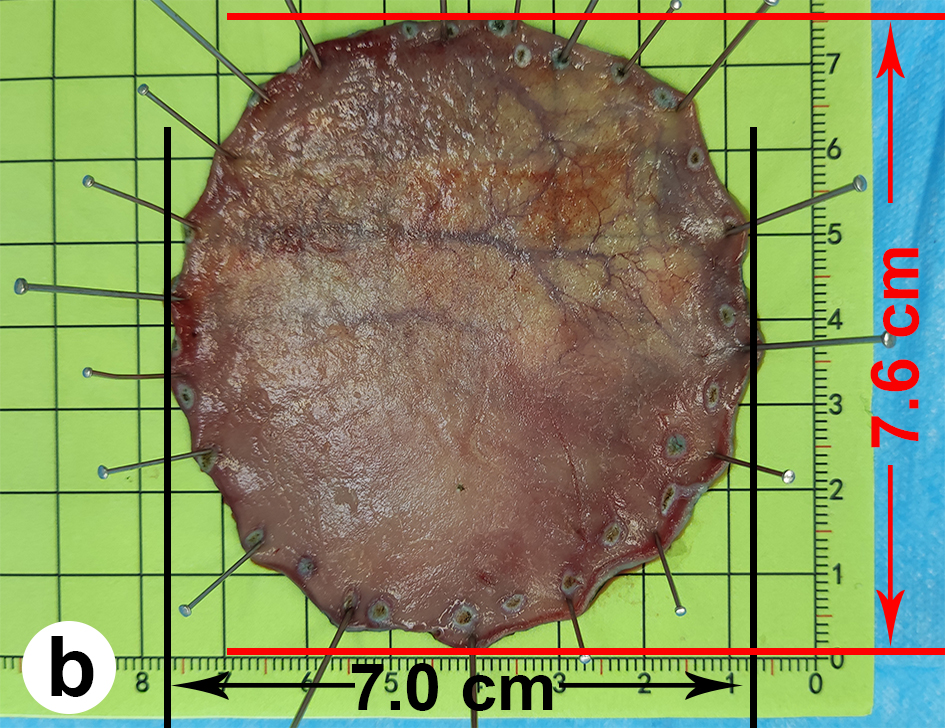


**Figure S1. Representative images of setting and measuring of defect size.** **a:** setting of the sizes of ESD using alcohol disinfected pig hair with given length; **b:** measuring size of the resected mucosal lesion.

**
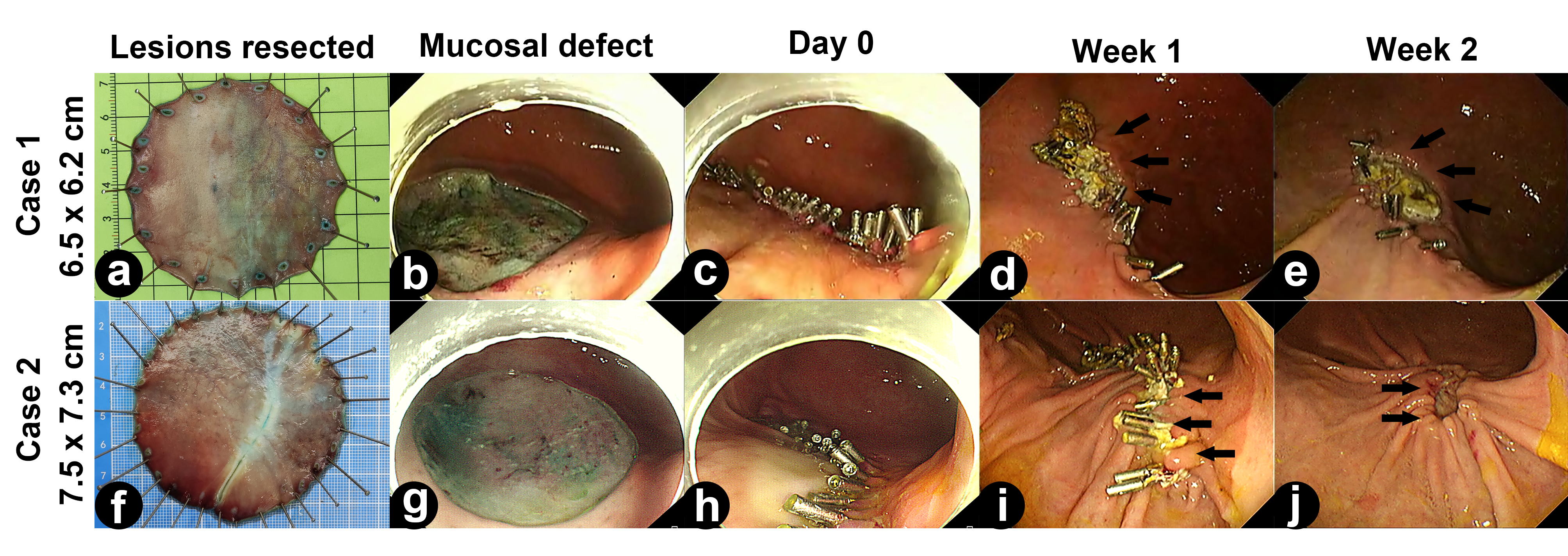
**

**Figure S2. Representative images of the two pigs with partial wound dehiscence.** All the two mucosal defects were successfully closed after surgery (c and h). The wound dehiscence was observed from middle to one side of the wound (black arrows) one week after surgery (d and i) and turned into natural ulcer healing two weeks after surgery (e and j).

**Supplementary video**

**Video S1. A representative case of using the twin-grasper to readdress the curly and sunk mucosal margins.**

**Video S2. A representative case of the new closure technique in the study.**
